# Association of variations in the *CAT* and prognosis in lung cancer patients with platinum-based chemotherapy

**DOI:** 10.3389/fphar.2023.1119837

**Published:** 2023-03-09

**Authors:** Jia-Si Liu, Jun-Yan Liu, Qi Xiao, Xiang-Ping Li, Juan Chen, Zhao-Qian Liu

**Affiliations:** ^1^ Department of Clinical Pharmacology, Xiangya Hospital, Central South University, Changsha, China; ^2^ Human Key Laboratory of Pharmacogenetics, and National Clinical Research Center for Geriatric Disorders, Xiangya Hospital, Central South University, Changsha, China; ^3^ Institute of Clinical Pharmacology, Engineering Research Center for Applied Technology of Pharmacogenomics of Ministry of Education, Central South University, Changsha, China; ^4^ Department of Orthopaedics, Xiangya Hospital, Central South University, Changsha, China; 5Department of Pharmacy, Xiangya Hospital, Central South University, Changsha, China

**Keywords:** lung cancer, platinum-based chemotherapy, snps, cat, ATM, ATR, precious medicine

## Abstract

**PURPOSE:** To explore the relationship between *ATM, ATR* and *CAT* polymorphisms and prognosis of lung cancer patients received platinum-based chemotherapy.

**METHODS:** 404 patients with lung cancer who received platinum-chemotherapy were enrolled and DNA typing was performed. Cox regression analysis and stratification analyses was performed to assess relationships between OS and PFS with SNPs genotypes. The prognosis of lung adenocarcinomaand squamous cell carcinomapatients was analyzed with The Cancer Genome Atlas (TCGA) database according to the grouping of *CAT* expression.

**RESULTS:** *CAT* rs769217 was significantly related to PFS of patients with lung cancer who received platinum-chemotherapy. In the Additive model, rs769217 was associated with PFS (HR = 0.747, 95% CI = 0.581–0.960, *p* = 0.023). In the Dominant model, CT and TT genotypes led to lung cancer progression 0.738 times more than CC genotype. In stratification analyses of association between *CAT* rs769217 polymorphisms and PFS, the HR of patients at stage IV in additive model was 0.73, and HR was 0.745 (*p* = 0.034) in dominant model. For OS analyses, HR was 0.672 in the older lung cancer patients (>55 years old) in additive model. Meanwhile, in the Dominant model, it was found that the older patients with CT and TT genotypes had better prognosis, and the risk of death after receiving platinum-based chemotherapy was 0.692 times that of patients with CC genotype (*p* = 0.037). TCGA data shows that LUAD patients with high *CAT* expression have longer OS (*p* = 0.020).

**CONCLUSION:** *CAT* rs769217 is significantly related to PSF of platinum-based chemotherapy in lung cancer patients and may be a biomarker for predicting the prognosis of lung cancer patients with platinum-based chemotherapy.

## 1 Introduction

Lung cancer is still the main cause of cancer death in the worldwide. There were an estimated 2,206,771 new cases and 1,796,144 cancer deaths of lung ancer worldwide in 2020 according to GLOBOCAN 2020[1]. Chemotherapeutic drugs have been widely used in the treatment of cancer disease for about 70 years. The development of new treatments has not hindered their use, and oncologists still prescribe them routinely, alone or in combination with other antineoplastic agents[2]. Platinum-based chemotherapy, as a conventional treatment, is usually used in combination with immunotherapy as a first-line treatment for most patients with metastatic non-small-cell lung cancer[3]. Platinum drugs can lead to an intrastrand or interstrand cross-linkage by interacting with DNA, thereby activating cellular processes leading to apoptosis[4]. There are many factors that affect the sensitivity of cancer cells to platinum chemotherapeutic drugs[5–7]. Based on previous studies and publication research, we hypothesized that *ATM* (ataxia telangiectasia mutant gene), *ATR* (ataxia telangiectasia and Rad3 related) and *CAT* (catalase) polymorphisms may be related to the prognosis of lung cancer patients receiving platinum-chemotherapy[8–10].

ATM and ATR kinases were the key mediators of DNA damage response (DDR), which induce cell cycle arrest and facilitate DNA repair *via* their downstream targets[11–13]. Obviously, here is consequently a strong rationale that *ATM* and *ATR* may be potential targets to affect platinum chemosensitivity[14,15]. Research shows that functional loss of ATR leads to abrogation of the DNA damage-induced G2/M cell cycle arrest and sensitization of cells to a variety of DNA damaging chemotherapeutic agents[11]. *ATM* and *ATR* SNPs may regulate kinase to enhance the DNA-damaging effect of Pt-based chemotherapy in cancer cells[9,16,17].

Platinum-based chemotherapy causes cancer cells death through inducing oxidative stress to highly toxic level[18]. Emerging data indicate that abnormally high ROS(reactive oxygen species) levels may instigate chemoresistance[19]. ROS can provide metabolic reprograming, promoting PGC-1α expression and mitochondrial mass that are in favor of cisplatin resistance in non-small cell lung cancer[20,21]. Therefore, the genetic polymorphism of oxidative stress related genes is likely to affect the platinum chemosensitivity. CAT are well studied enzymes that play critical roles in protecting cells against the toxic effects of hydrogen peroxide, which is a key enzyme in the metabolism of H_2_O_2_, reducing the production of ROS in cells[22]. Studies have shown that *CAT* rs769218 is related to the prognosis of gastric cancer patients receiving platin and fluorouracil-based adjuvant therapy, but the correlation between the prognosis of lung cancer patients and *CAT* polymorphism has not been reported[18]. Herein, we investigated the association of potentially functional SNPs in *ATM, ATR* and *CAT* with platinum-based chemotherapy outcome of lung cancer patients.

## 2 Materials and methods

### 2.1 Study population and data collection

All patients in this study were recruited from Xiangya Hospital of Central South University and Hunan Cancer Hospital (Changsha, China), from 2012 to 2019. All patients were diagnosed with NSCLC by histopathological examination and confirmed the absence of driver genetic alterations that could be targeted. All patients received first-line platinum-based chemotherapy regimens for two to six cycles: cisplatin (75 mg/m^2^) or carboplatin (AUC 5), which were both administered on Day 1 every 3 weeks, in combination with pemetrexed (500 mg/m^2^) on Day 1 every 3 weeks, gemcitabine (1,250 mg/m^2^) on Days 1 and 8 every 3 weeks, paclitaxel (175 mg/m^2^) on Day 1 every 3 weeks, docetaxel (75 mg/m2) on Day 1 every 3 weeks, or navelbine (25 mg/m^2^) on Days 1 and 8 every 3 weeks. Patients did not undergo surgery, targeted therapy, radiotherapy, or other anti-tumor therapy before chemotherapy. A physical examination as well as a detailed inquiry into each patient’s medical history was carried out. Patients with serious concomitant diseases that might greatly affect their physical condition were excluded. The study protocol was approved by the Ethics Committee of Institute of Clinical Pharmacology, Central South University (Changsha, China), and all subjects were provided with written informed consents. We applied this study for clinical admission in the Chinese Clinical Trial Register (registration number: ChiCTR-RO-12002873)[23].

### 2.2 DNA extraction and genotyping, SNP selection

In the morning, 5 mL blood of lung cancer patients undergoing chemotherapy was collected. After centrifugation at 4000rpm, plasma was separated and stored at −20 °C. DNA was extracted using Genomic DNA Purification Kit Genomic (Promega, Madison, WI, United States) and stored at −20°C according to the instructions. Each DNA sample was genotyped by Sequenom Mass Array Genotype Platform (Sequenom, SanDiego, CA, United States). After polymerase chain reaction (PCR), the product was purified by resin and analyzed by the Mass Array system (Sequenom)[24]. Then we used Plink (version 1.9, http://pngu.mgh.harvard.edu/purcell/plink/)to detect and control the quality of SNPs data, requiring that the MAF (Minor allele frequency) ≥0.05, the call rate≥90%, and conform to Hardy-Weinberg equilibrium. Finally, we selected 7 SNPs of ATR, ATM, and *CAT* for follow-up (Table 1).

**TABLE 1:** Association of comorbidities with CAT genotypes in lung cancer patients.

Comorbidities	Statue	*CAT* rs769217 genotype			*p*-Value
		CC	CT	TT	
Hypertension	Yes	17 (4.3)	29 (7.34)	7 (1.77)	0.389
	No	98 (24.81)	171 (43.29)	73 (18.48)	
Diabetes mellitus	Yes	6 (1.52)	11 (2.78)	8 (2.03)	0.318
	No	109 (27.59)	189 (47.85)	72 (18.23)	
COPD	Yes	17 (4.30)	32 (8.10)	17 (4.30)	0.457
	No	98 (24.81)	168 (42.53)	63 (15.95)	
coronary heart disease	Yes	1 (0.25)	10 (2.53)	2 (0.51)	0.137
	No	114 (28.86)	190 (48.10)	78 (19.75)	
pulmonary tuberculosis	Yes	7 (1.77)	4 (1.01)	4 (1.01)	0.144
	No	108 (27.34)	196 (49.62)	76 (19.24)	
Chronic viral hepatitis	Yes	4 (1.01)	13 (3.29)	9 (2.28)	0.111
	No	111 (28.10)	187 (47.34)	71 (17.97)	

### 2.3 Statistical analysis

OS (Overall survival) was defined as the time from treatment initiation until either the date of death, the date of last follow-up or the date of end of analysis. PFS (Progression-free survival) was defined as the time from treatment initiation until disease progression or death, whichever occurred first[25]. Kaplan-Meier analysis was used to calculate the median survival time of lung cancer patients undergoing chemotherapy. COX proportional hazards regression analysis was used to analyze the correlation between genotypes and prognosis in each Genetic Model after adjusting for age, sex, smoking, histology, and stage confounding factors. Stratified analysis was used to analyze the correlation between SNPs in each layer and the prognosis of lung cancer patients with platinum-based chemotherapy, so as to control the influence of covariates on the research results to a certain extent. Hazard ratio (HR) was used to evaluate the degree of effect between genotype and patient prognosis. 95% confidence interval (95% CI) is the range of HR estimated by 95% probability. All the *p*-values were two-sided, *p* < 0.05 were supposed to be significant. All the above analyses were performed by The SPSS version 25.0 (SPSS Inc., Chicago, IL, United States). UCSC Xena (https://xena.ucsc.edu/kaplan-survival-analysis/) was used to analyze the prognosis of LUAD and LUSC patients in TCGA database.

## 3 Results

### 3.1 Characteristics and prognosis of lung cancer patients

A total of 404 lung cancer patients received platinum-based chemotherapy were included in this research. There were 175 patients with age ≤55 years old, accounting for 43.32%. Most of the patients were male, accounting for 77.48% (313). 40.10% (162) patients have the habit of smoking. 97.52% of the patients (394) were in stage III or IV. 50% of the patients (202) were adenocarcinoma and 42.33% (171) were small cell carcinoma (Table 2).

**TABLE 2:** Association of 7 SNPs polymorphisms and PFS.

Gene	Polymorphism	Genotype	MPFS (year)	Additive			Dominant			Recessive		
				Genotype	HR (95%CI)	*p*-value	Genotype	HR (95%CI)	*p*-value	Genotype	HR (95%CI)	*p*-value
*ATM*	rs228589	AA	1.855	AA	REF	0.409	AA	REF		TT	REF	
		AT	2.984	AT	1.133 (0.837–1.534)	0.419	AT + TT	0.950 (0.716–1.261)	0.722	AT + AA	0.880 (0.694–1.116)	0.293
		TT	4.2199	TT	0.96 (0.698–1.320)	0.800						
*ATR*	rs4585	GG	4.195	GG	REF	0.641	GG	REF		TT	REF	
		GT	3.014	GT	1.116 (0.865–1.440)	0.397	GT + TT	0.924 (0.727–1.174)	0.516	GT + GG	1.062 (0.793–1.421)	0.687
		TT	1.836	TT	1.003 (0.723–1.392)	0.984						
*ATR*	rs2227928	AA	3.452	AA	REF	0.513	AA	REF		GG	REF	
		AG	3.003	AG	1.164 (0.883–1.535)	0.283	AG + GG	0.892 (0.688–1.157)	0.391	AG + AA	1.057 (0.810–1.378)	0.684
		GG	4.381	GG	1.043 (0.756–1.438)	0.799						
*ATR*	rs2229032	CC	3.164	CC	REF	0.533	CC	REF		TT	REF	
		CT	2.151	CT	1.142 (0.829–1.571)	0.416	CT + TT	1.098 (0.802–1.503)	0.559	CT + CC	1.740 (0.427–7.090)	0.44
		TT	5.337	TT	0.585 (0.144–2.387)	0.455						
*CAT*	rs564250	TT	4.775	TT	REF	0.075	TT	REF		CC	REF	
		TC	3.8	TC	1.004 (0.481–2.095)	0.992	TC + CC	1.221 (0.598–2.492)	0.583	TC + TT	0.757 (0.595–0.962)	0.023
		CC	3.003	CC	1.326 (0.648–2.714)	0.44						
*CAT*	rs769217	CC	2.164	CC	REF	0.042	CC	REF		TT	REF	
		CT	3.8	CT	0.747 (0.581–0.960)	0.023	CT + TT	0.738 (0.582–0.936)	0.012	CT + CC	1.159 (0.873–1.539)	0.308
		TT	2.764	TT	0.715 (0.518–0.987)	0.041						
*CAT*	rs7943316	AA	3.066	AA	REF	0.812	AA	REF		TT	REF	
		AT	3.425	AT	1.049 (0.828–1.327)	0.693	AT + TT	1.063 (0.850–1.329)	0.594	AT + AA	0.908 (0.626–1.316)	0.609
		TT	2.307	TT	1.126 (0.764–1.661)	0.547						

### 3.2 Association of the *CAT* rs769217 polymorphisms and PFS in lung cancer patients with platinum-based chemotherapy

**FIGURE 1:**
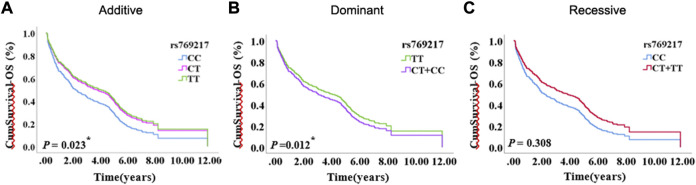
The Kaplan-Meier plot of COX proportional hazards regression analysis, **(A)** Additive model, **(B)** Dominant model, **(C)** Recessive model.

After excluding the effects of age, sex, smoking status, stage, and histology type, we used COX proportional hazards regression analysis to analyze the relationship between SNPs and patient prognosis, and found that *CAT* rs769217 was significantly related to PFS. In the additive model, rs769217 was associated with PFS, HR = 0.747, 95% CI = 0.581–0.960, *p* = 0.023. In the dominant model, CT + TT genotypes led to lung cancer progression 0.738 times more than CC genotype. In the recessive model, the *p*-value > 0.05, which is not significant (Table 3). However, the Kaplan-Meier plot still shows that the PFS of patients with CT and TT genotypes is longer than that of patients with CC genotype (Figure 1). The lung cancer patients’ comorbidities are not associated with *CAT* genotypes (Supplementary Table S1). The correlation of other SNPs with PFS and OS is shown in Supplementary Table S2, S3.

**TABLE 3:** Association of 7 SNPs polymorphisms and OS.

Gene	Polymorphism	Genotype	Mos (year)	Additive			Dominant			Recessive		
				Genotype	HR (95%CI)	*p*-value	Genotype	HR (95%CI)	*p*-value	Genotype	HR (95%CI)	*p*-value
*ATM*	rs228589	AA	3.186	AA	REF	0.959	AA	REF		TT	REF	
		AT	4.268	AT	0.955 (0.689–1.325)	0.785	AT + TT	1.047 (0.768–1.427)	0.771	AT + AA	1.013 (0.789–1.300)	0.922
		TT	4.671	TT	0.955 (0.679–1.352)	0.794						
*ATR*	rs4585	GG	4.381	GG	REF	0.652	GG	REF		TT	REF	
		GT	4.392	GT	0.915 (0.701–1.195)	0.514	GT + TT	1.055 (0.820–1.358)	0.675	GT + GG	0.900 (0.659–1.230)	0.508
		TT	3.186	TT	1.053 (0.741–1.495)	0.775						
*ATR*	rs2227928	AA	3.83	AA	REF	0.428	AA	REF		GG	REF	
		AG	4.115	AG	0.821 (0.610–1.105)	0.194	AG + GG	0.845 (0.640–1.116)	0.235	AG + AA	0.983 (0.742–1.301)	0.904
		GG	4.679	GG	0.893 (0.636–1.254)	0.512						
*ATR*	rs2229032	CC	4.268	CC	REF	0.687	CC	REF		TT	REF	
		CT	3.449	CT	1.15 (0.825–1.604)	0.409	CT + TT	1.154 (0.833–1.598)	0.389	CT + CC	0.826 (0.202–3.369)	0.789
		TT	1.627	TT	1.232 (0.302–5.030)	0.772						
*CAT*	rs564250	TT	3.066	TT	REF	0.178	TT	REF		CC	REF	
		TC	3.942	TC	0.653 (0.313–1.363)	0.257	TC + CC	0.764 (0.375–1.557)	0.459	TC + TT	0.823 (0.639–1.058)	0.129
		CC	4.268	CC	0.820 (0.401–1.677)	0.586						
*CAT*	rs769217	CC	3.888	CC	REF	0.241	CC	REF		TT	REF	
		CT	4.658	CT	0.800 (0.610–1.048)	0.105	CT + TT	0.832 (0.644–1.075)	0.159	CT + CC	0.927 (0.690–1.247)	0.618
		TT	3.205	TT	0.931 (0.662–1.310)	0.683						
*CAT*	rs7943316	AA	4.392	AA	REF	0.806	AA	REF		TT	REF	
		AT	4.049	AT	1.024 (0.800–1.312)	0.85	AT + TT	0.997 (0.788–1.261)	0.978	AT + AA	1.143 (0.754–1.732)	0.53
		TT	4.671	TT	0.885 (0.575–1.361)	0.577						

### 3.3 Stratification analyses of association between *CAT* rs769217 polymorphisms and PFS

**FIGURE 2:**
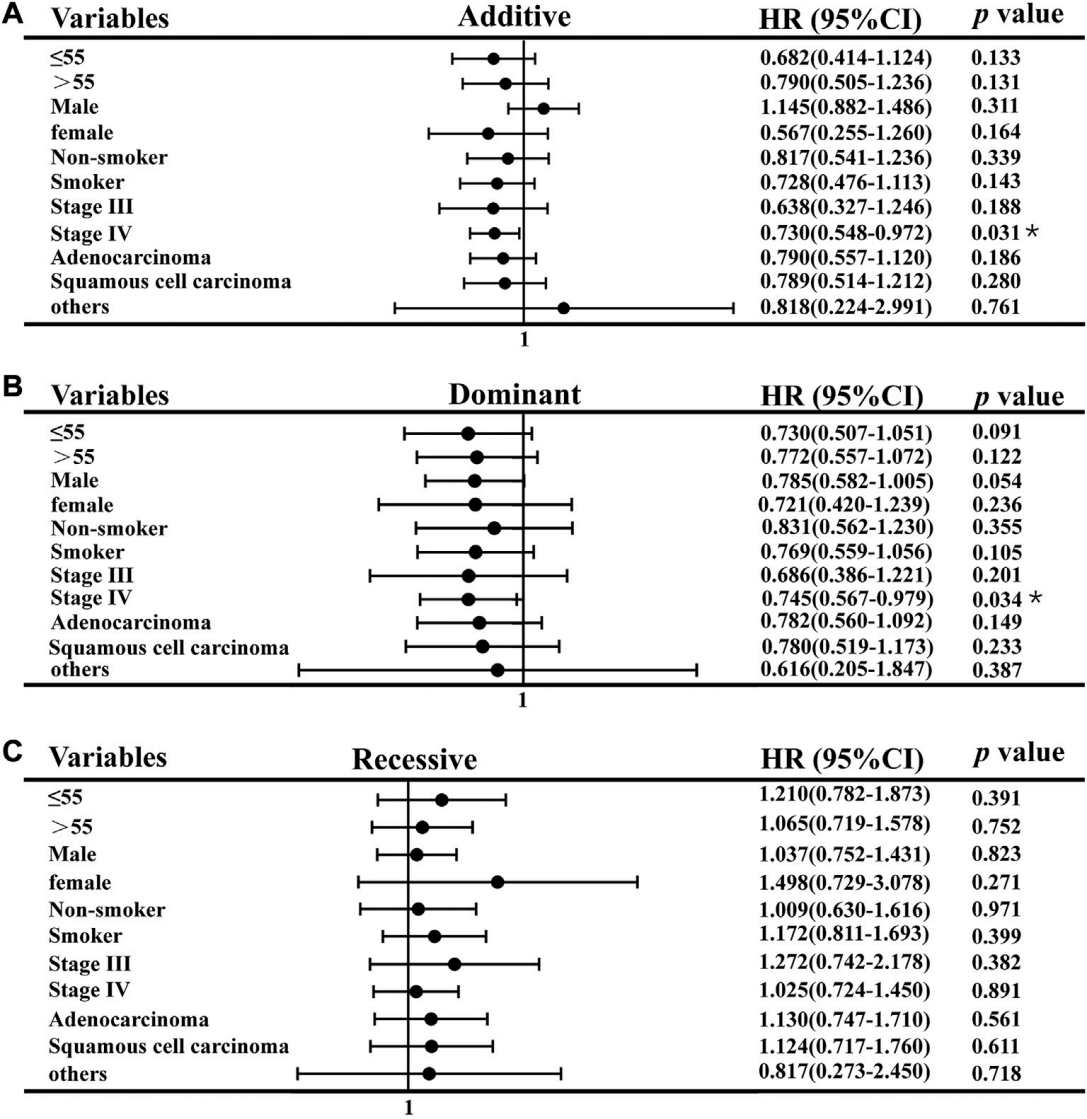
Association of CAT rs769217 and PFS in Stratification analyses, **(A)** additive model, **(B)** Dominant model, **(C)** Recessive model. p* <0.05, p**< 0.01.

**FIGURE 3:**
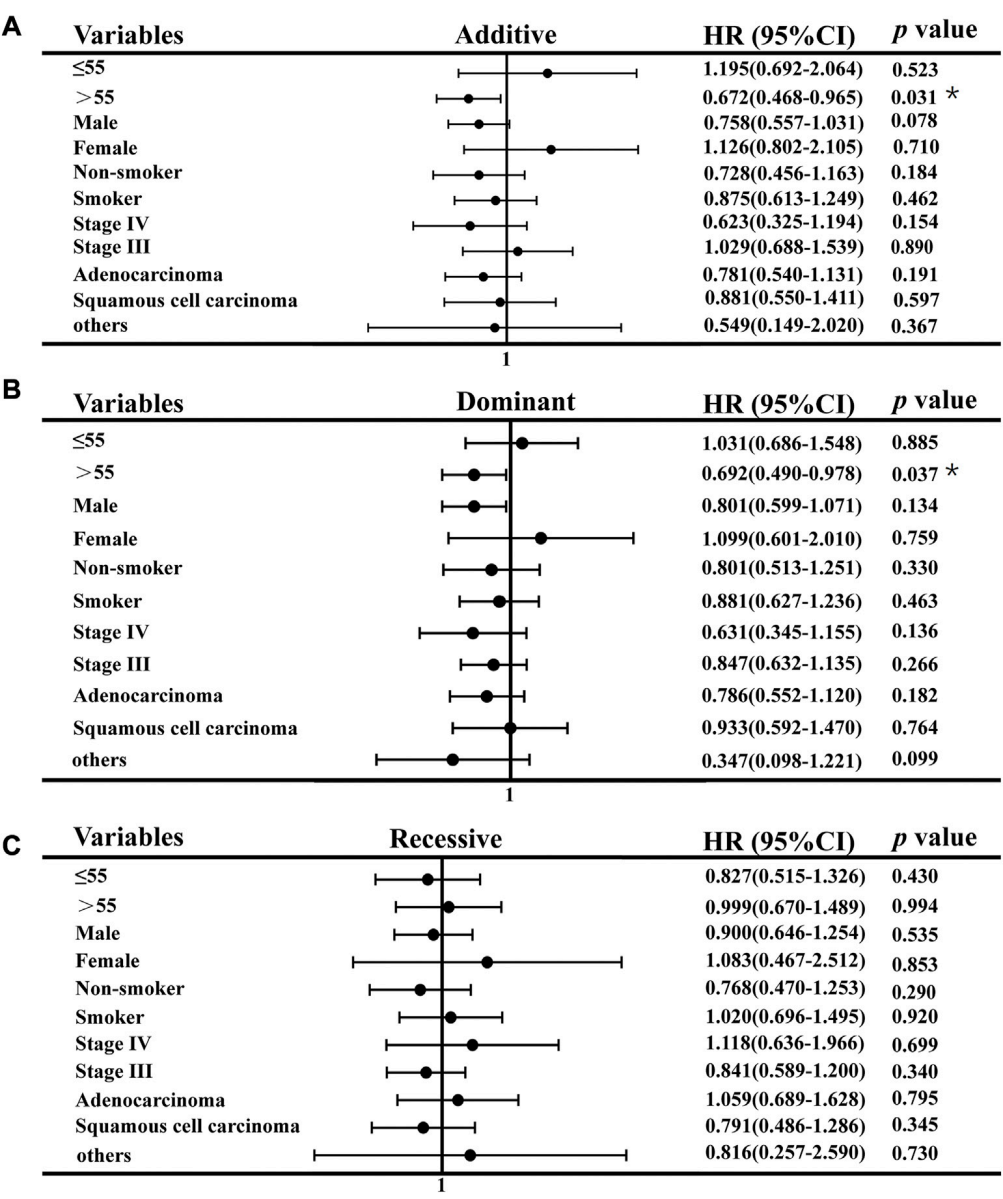
Association of CAT rs769217 and OS in Stratification analyses, **(A)** additive model, **(B)** Dominant model, **(C)** Recessive model. p* <0.05, p**< 0.01.

We used stratification analysis to stratify the clinical data of lung cancer patients receiving platinum-based chemotherapy according to age, sex, smoking status, histology, and stage, and then calculated the association between SNP rs769217 polymorphism and prognosis. In the correlation analysis between SNP polymorphism and PFS, the HR of patients at stage IV in the Additive model was 0.730, which indicates patients with CT genotype are 0.73 times more likely to progress than those with CC genotype, and the prognosis of patients with CT genotype is better (*p* = 0.031). In the Dominant model, the HR of patients in stage IV was 0.745, *p* = 0.034 (Figure 2). In the correlation analysis between SNP polymorphism and OS, HR = 0.672, *p* = 0.031 of the older lung cancer patients (>55 years old) was found in the Additive model. Meanwhile, in the Dominant model, it was found that the older patients with CT and TT genotype had better prognosis, and the risk of death after receiving platinum-based chemotherapy was 0.692 times that of patients with CC genotype (*p* = 0.037) (Figure 3).

## 4 Discussion

In 2019, about 2 million people worldwide died from lung cancer, more than any other cancer[26]. Annually, approximately 631,000 deaths were reported because of lung cancer according to Chinese national statistics[27]. Compared with the decline of the incidence in some western countries, the incidence of lung cancer in China is still rising, which is a major public health problem[28]. Chemotherapy and immunotherapy are common therapeutic methods in clinic. Immunotherapy has incomparable advantages over traditional anti-tumor therapy, which can prolong PFS and OS. The current reality, however, is that the majority of patients often cannot benefit from it because of low tumor mutation burden, or other reasons, or terminate the treatment due to serious adverse reactions[29,30]. Therefore, platinum-based chemotherapy, as an effective anti-tumor therapy, is still the first-line drug regimen for lung cancer[23]. After platinum chemotherapeutic drugs enter the tumor cell, a series of chemical reactions occur in the cytoplasm. Platinum binds to DNA by forming intra- and inter-stranded crosslinks, changing the DNA structure, and causing DNA damage, so as to achieve the purpose of anti-tumor[31]. In addition, platinum-based chemotherapy was shown to upregulate tumor cell expression of PD-L1 and has immunostimulatory properties as well, which plays an anti-tumor role in coordination with immunotherapy[32–35]. Genetic abnormalities could influence chemosensitivity, the development of predictive markers to identify patients who will derive significant benefit with minimal toxicity from chemotherapy is a continuing challenge in lung cancer research[36,37]. However, the genetic underpinnings of platinum sensitivity remain poorly understood[38].

DNA is usually considered as the main target of platinum chemotherapeutic drugs, but the cisplatin used *in vitro* studies lead to acute apoptosis that involves induction of oxidative stress but is largely DNA damage-independent[39]. Cellular exposure to cisplatin causes direct damage to mtDNA resulting in a reduction of mitochondrial protein synthesis, impairment of electron transport chain function, and subsequently, increases in intracellular ROS levels, ultimately promoting cell death[40]. However, due to the double-edged sword role of ROS in cancer as a pro-survival or pro-death mechanism, ROS can result in platinum-chemotherapy resistance[21]. ROS can provide metabolic reprograming, promoting PGC-1α expression and mitochondrial mass that are in favor of cisplatin resistance in non-small cell lung cancer[20].

**FIGURE 4:**
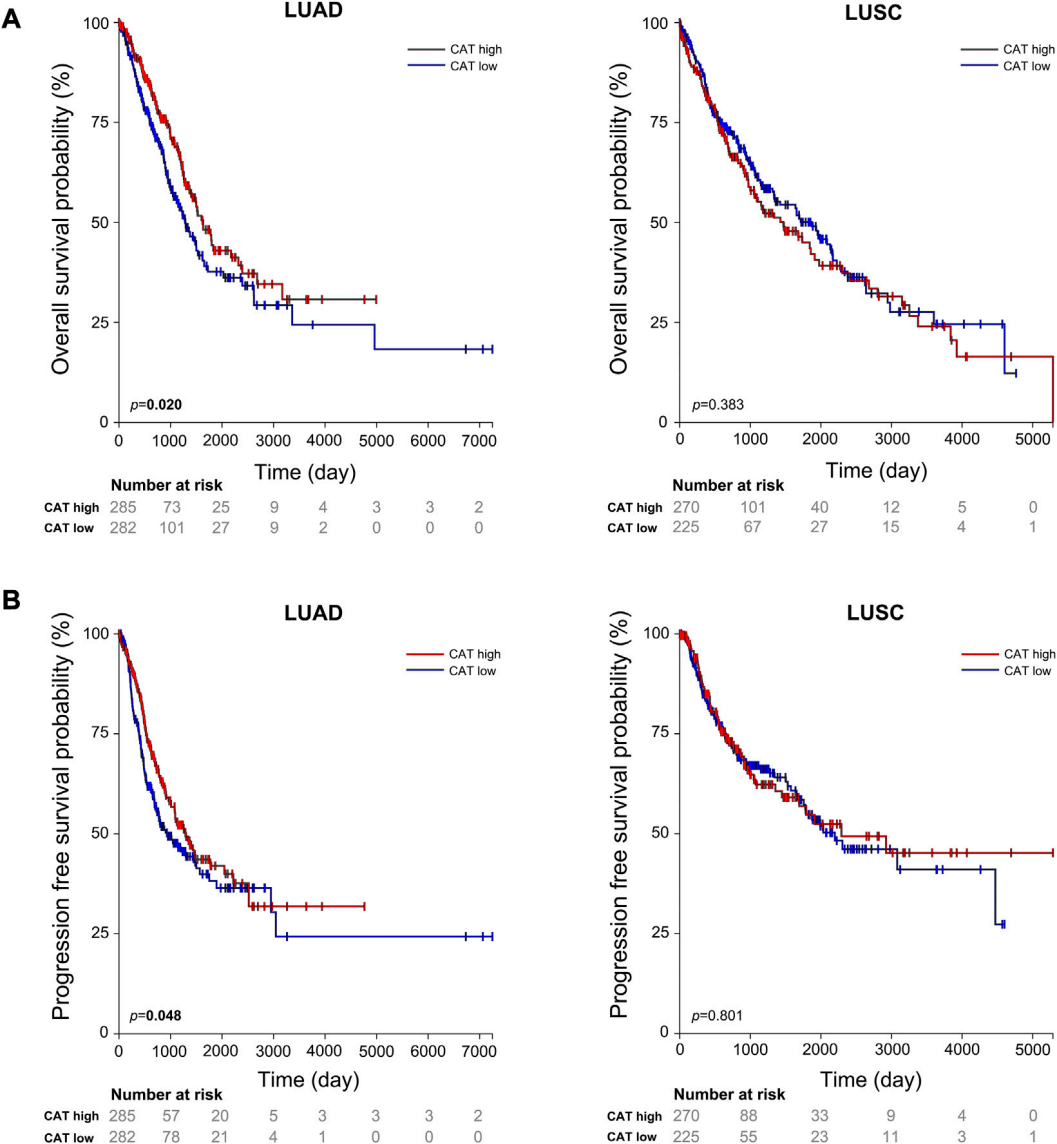
Association of the expression of CAT with lung cancer OS **(A)** and PFS **(B)** in TCGA LUAD and LUSC patients.

Our results suggest that *CAT* rs769217 may affect the PFS of lung cancer patients receiving platinum-based chemotherapy (Figure 1). *CAT* gene encodes catalase, which can regulate reactive oxygen species (ROS), is a key antioxidant enzyme in the bodies defense against oxidative stress[41,42]. Previous studies showed that as compared to normal tissues of the same origin, the expression of CAT in tumors changed[43–45]. Two meta-analyses pointed out a correlation exists between *CAT* rs1001179 polymorphism and prostate cancer[46,47]. At the same time, the expression of CAT in tumor cells can affect their sensitivity to chemotherapy drugs[48–50]. There are definite experimental results proving that *CAT* rs769217 can affect the prognosis of patients with biliary tr an act cancer (BTC), that knockdown of *CAT* induced chemoresistance through elevation of ROS level and activation of Nrf2-ABCG2 pathway in BTC cell lines[51]. But the impact of *CAT* polymorphism on the prognosis of patients with lung cancer receiving platinum chemotherapy has not been reported. *CAT* polymorphisms can affect the expression of *CAT* mRNA in tumor tissues[51], and according to TCGA data analysis, the OS and PFS of LUAD patients with high *CAT* expression is significantly longer (*p* = 0.020, *p* = 0.048 respectively; Figure 4). We speculate that compared with CC genotype, patients with lung cancer who carry TT genotype and receive platinum-chemotherapy have higher *CAT* expression in tumor cells, thus regulating ROS and making tumor cells sensitive to platinum-chemotherapy, but the specific mechanism needs to be further explored. Therefore, *CAT* can be used as a potential target to enhance the sensitivity of platinum-chemotherapy, nanocarriers of platinum and CAT enhance the cytotoxicity of drug resistant cancer cells[52]. In addition, we found that compared with LUAD patients, the prognosis of LUSC patients was not associated with *CAT* expression **(** Figure 4). The expression of most DNA repair genes in LUSC tumor cells is upregulated[53]. Lung cancer patients with higher DNA repair capacity had elevated chemoresistance[54]. Therefore, we speculate that the effect of these DNA repair genes on the efficacy of platinum drugs is far greater than that of *CAT* genes. In conclusion, our study showed that *CAT* rs769217 is significantly related to PSF of platinum-based chemotherapy in lung cancer patients. *CAT* rs769217 may be a biomarker for predicting the prognosis of lung cancer patients with platinum-based chemotherapy.

## Data Availability

The publicly available data sets that supported this study are available from OMIX under accessions OMIX002961.
