# Supplementary material for: Association of variations in the CAT and prognosis in lung cancer patients with platinum-based chemotherapy
Source: Front Pharmacol. 2023 Mar 9;14:1119837. doi: 10.3389/fphar.2023.1119837 (PMC10033691; doi:10.3389/fphar.2023.1119837)
Supplement: Supplementary file 1 [file Table1.docx]

**Table S1.** Association of **comorbidities with *CAT* genotypes in lung cancer patients**

| **Comorbidities** | **Statue** | ***CAT* rs769217 genotype** | | | ***P* value** |
| --- | --- | --- | --- | --- | --- |
|  |  | **CC** | **CT** | **TT** |  |
| Hypertension | Yes | 17(4.3) | 29(7.34) | 7(1.77) | 0.389 |
|  | No | 98(24.81) | 171(43.29) | 73(18.48) |  |
| Diabetes mellitus | Yes | 6(1.52) | 11(2.78) | 8(2.03) | 0.318 |
|  | No | 109(27.59) | 189(47.85) | 72(18.23) |  |
| COPD | Yes | 17(4.30) | 32(8.10) | 17(4.30) | 0.457 |
|  | No | 98(24.81) | 168(42.53) | 63(15.95) |  |
| coronary heart disease | Yes | 1(0.25) | 10(2.53) | 2(0.51) | 0.137 |
|  | No | 114(28.86) | 190(48.10) | 78(19.75) |  |
| pulmonary tuberculosis | Yes | 7(1.77) | 4(1.01) | 4(1.01) | 0.144 |
|  | No | 108(27.34) | 196(49.62) | 76(19.24) |  |
| Chronic viral hepatitis | Yes | 4(1.01) | 13(3.29) | 9(2.28) | 0.111 |
|  | No | 111(28.10) | 187(47.34) | 71(17.97) |  |
