# Supplementary material for: Association of variations in the CAT and prognosis in lung cancer patients with platinum-based chemotherapy
Source: Front Pharmacol. 2023 Mar 9;14:1119837. doi: 10.3389/fphar.2023.1119837 (PMC10033691; doi:10.3389/fphar.2023.1119837)
Supplement: Supplementary file 2 [file Table2.docx]

**Table S2. Association of 7 SNPs polymorphisms and PFS**

***ATM*:** ataxia telangiectasia mutant. ***ATR*:** ataxia telangiectasia and Rad3 related. ***CAT*:** catalase. **MPFS:** median survival time of progression-free survival**. HR:** Hazard ratio**.**

**95%CI:** 95% confidence interval.

| **Gene** | **Polymorphism** | **Genotype**  **(number)** | **MPFS (year)** | **Additive** | | | **Dominant** | | | **Recessive** | | |
| --- | --- | --- | --- | --- | --- | --- | --- | --- | --- | --- | --- | --- |
|  |  |  |  | **Genotype** | **HR (95%CI)** | ***p* value** | **Genotype** | **HR (95%CI)** | ***p* value** | **Genotype** | **HR (95%CI)** | ***p* value** |
| *ATM* | rs228589 | AA (80) | 1.855 | AA | REF | 0.409 | AA | REF |  | TT | REF |  |
|  |  | AT (181) | 2.984 | AT | 1.133(0.837-1.534) | 0.419 | AT+TT | 0.950(0.716-1.261) | 0.722 | AT+AA | 0.880(0.694-1.116) | 0.293 |
|  |  | TT (134) | 4.2199 | TT | 0.96(0.698-1.320) | 0.800 |  |  |  |  |  |  |
| *ATR* | rs4585 | GG (130) | 4.195 | GG | REF | 0.641 | GG | REF |  | TT | REF |  |
|  |  | GT (182) | 3.014 | GT | 1.116(0.865-1.440) | 0.397 | GT+TT | 0.924(0.727-1.174) | 0.516 | GT+GG | 1.062(0.793-1.421) | 0.687 |
|  |  | TT (76) | 1.836 | TT | 1.003(0.723-1.392) | 0.984 |  |  |  |  |  |  |
| *ATR* | rs2227928 | AA (103) | 3.452 | AA | REF | 0.513 | AA | REF |  | GG | REF |  |
|  |  | AG (191) | 3.003 | AG | 1.164(0.883-1.535) | 0.283 | AG+GG | 0.892(0.688-1.157) | 0.391 | AG+AA | 1.057(0.810-1.378) | 0.684 |
|  |  | GG (93) | 4.381 | GG | 1.043(0.756-1.438) | 0.799 |  |  |  |  |  |  |
| *ATR* | rs2229032 | CC (338) | 3.164 | CC | REF | 0.533 | CC | REF |  | TT | REF |  |
|  |  | CT (54) | 2.151 | CT | 1.142(0.829-1.571) | 0.416 | CT+TT | 1.098(0.802-1.503) | 0.559 | CT+CC | 1.740(0.427-7.090) | 0.44 |
|  |  | TT (3) | 5.337 | TT | 0.585(0.144-2.387) | 0.455 |  |  |  |  |  |  |
| *CAT* | rs564250 | TT (10) | 4.775 | TT | REF | 0.075 | TT | REF |  | CC | REF |  |
|  |  | TC (124) | 3.8 | TC | 1.004(0.481-2.095) | 0.992 | TC+CC | 1.221(0.598-2.492) | 0.583 | TC+TT | 0.757(0.595-0.962) | **0.023** |
|  |  | CC (264) | 3.003 | CC | 1.326(0.648-2.714) | 0.44 |  |  |  |  |  |  |
| *CAT* | rs7943316 | AA (193) | 3.066 | AA | REF | 0.812 | AA | REF |  | TT | REF |  |
|  |  | AT (163) | 3.425 | AT | 1.049(0.828-1.327) | 0.693 | AT+TT | 1.063(0.850-1.329) | 0.594 | AT+AA | 0.908(0.626-1.316) | 0.609 |
|  |  | TT (38) | 2.307 | TT | 1.126(0.764-1.661) | 0.547 |  |  |  |  |  |  |
