# Supplementary material for: Association of variations in the CAT and prognosis in lung cancer patients with platinum-based chemotherapy
Source: Front Pharmacol. 2023 Mar 9;14:1119837. doi: 10.3389/fphar.2023.1119837 (PMC10033691; doi:10.3389/fphar.2023.1119837)
Supplement: Supplementary file 3 [file Table3.docx]

**Table S3. Association of 7 SNPs polymorphisms and OS**

***ATM***: ataxia telangiectasia mutant. ***ATR***: ataxia telangiectasia and Rad3 related. ***CAT***: catalase. **MOS:** median survival time of overall survival**. HR:** Hazard ratio**.**

| **Gene** | **Polymorphism** | **Genotype**  **(number)** | **MOS (year)** | **Additive** | | | **Dominant** | | | **Recessive** | | |
| --- | --- | --- | --- | --- | --- | --- | --- | --- | --- | --- | --- | --- |
|  |  |  |  | **Genotype** | **HR (95%CI)** | ***p* value** | **Genotype** | **HR (95%CI)** | ***p* value** | **Genotype** | **HR (95%CI)** | ***p* value** |
| *ATM* | rs228589 | AA (60) | 3.186 | AA | REF | 0.959 | AA | REF |  | TT | REF |  |
|  |  | AT (149) | 4.268 | AT | 0.955(0.689-1.325) | 0.785 | AT+TT | 1.047(0.768-1.427) | 0.771 | AT+AA | 1.013(0.789-1.300) | 0.922 |
|  |  | TT (109) | 4.671 | TT | 0.955(0.679-1.352) | 0.794 |  |  |  |  |  |  |
| *ATR* | rs4585 | GG (106) | 4.381 | GG | REF | 0.652 | GG | REF |  | TT | REF |  |
|  |  | GT (149) | 4.392 | GT | 0.915(0.701-1.195) | 0.514 | GT+TT | 1.055(0.820-1.358) | 0.675 | GT+GG | 0.900(0.659-1.230) | 0.508 |
|  |  | TT (59) | 3.186 | TT | 1.053(0.741-1.495) | 0.775 |  |  |  |  |  |  |
| *ATR* | rs2227928 | AA (76) | 3.83 | AA | REF | 0.428 | AA | REF |  | GG | REF |  |
|  |  | AG (159) | 4.115 | AG | 0.821(0.610-1.105) | 0.194 | AG+GG | 0.845(0.640-1.116) | 0.235 | AG+AA | 0.983(0.742-1.301) | 0.904 |
|  |  | GG (76) | 4.679 | GG | 0.893(0.636-1.254) | 0.512 |  |  |  |  |  |  |
| *ATR* | rs2229032 | CC (271) | 4.268 | CC | REF | 0.687 | CC | REF |  | TT | REF |  |
|  |  | CT (48) | 3.449 | CT | 1.15(0.825-1.604) | 0.409 | CT+TT | 1.154(0.833-1.598) | 0.389 | CT+CC | 0.826(0.202-3.369) | 0.789 |
|  |  | TT (2) | 1.627 | TT | 1.232(0.302-5.030) | 0.772 |  |  |  |  |  |  |
| *CAT* | rs564250 | TT (213) | 3.066 | TT | REF | 0.178 | TT | REF |  | CC | REF |  |
|  |  | TC (100) | 3.942 | TC | 0.653(0.313-1.363) | 0.257 | TC+CC | 0.764(0.375-1.557) | 0.459 | TC+TT | 0.823(0.639-1.058) | 0.129 |
|  |  | CC (213) | 4.268 | CC | 0.820(0.401-1.677) | 0.586 |  |  |  |  |  |  |
| *CAT* | rs769217 | CC (93) | 3.888 | CC | REF | 0.241 | CC | REF |  | TT | REF |  |
|  |  | CT (165) | 4.658 | CT | 0.800(0.610-1.048) | 0.105 | CT+TT | 0.832(0.644-1.075) | 0.159 | CT+CC | 0.927(0.690-1.247) | 0.618 |
|  |  | TT (62) | 3.205 | TT | 0.931(0.662-1.310) | 0.683 |  |  |  |  |  |  |
| *CAT* | rs7943316 | AA (158) | 4.392 | AA | REF | 0.806 | AA | REF |  | TT | REF |  |
|  |  | AT (130) | 4.049 | AT | 1.024(0.800-1.312) | 0.85 | AT+TT | 0.997(0.788-1.261) | 0.978 | AT+AA | 1.143(0.754-1.732) | 0.530 |
|  |  | TT (29) | 4.671 | TT | 0.885(0.575-1.361) | 0.577 |  |  |  |  |  |  |
